# Prospective investigation of drug resistance: an approach to understanding and optimizing the clinical benefit of targeted agents in Ewing sarcoma

**DOI:** 10.18632/oncotarget.26465

**Published:** 2018-12-18

**Authors:** Kelly M. Bailey

**Affiliations:** Kelly M. Bailey: Department of Pediatrics, Division of Hematology/Oncology, University of Pittsburgh School of Medicine, Pittsburgh, Pennsylvania, United States of America

**Keywords:** Ewing sarcoma, drug resistance

Metastatic and relapsed Ewing sarcoma is a notoriously difficult to treat pediatric cancer. In recent decades, scientists have gained a better understanding of this tumor through identifying EWS/FLI1 (the most common fusion oncoprotein driving this cancer) and characterizing the impact of EWS/FLI1 on multiple facets of Ewing tumor cell biology [1]. Despite decades of scientific progress, finding candidate targeted agents to treat Ewing sarcoma has been challenging, as these tumors are genetically ‘quiet’, having one of the lowest mutational burdens of all cancers [2, 3]. To date, therapeutics aimed at targeting vulnerable pathways in Ewing sarcoma have not resulted in successful new treatments in the relapsed setting. Metastatic and therapy non-naive Ewing cells are adept at evading both conventional chemotherapy and targeted agents. Recently, the EWS/FLI1 fusion oncoprotein was shown to promote Ewing tumor cell susceptibility to PARP inhibition [4]. Despite this strong preclinical data, to date, PARP inhibitors have fallen short at demonstrating significant clinical efficacy in Ewing sarcoma. Heisey et al investigated the mechanisms of Ewing cell resistance to the PARP inhibitor olaparib and found that BCL2 and BCL2L1 (BCL-XL) are key mediators of PARP resistance in Ewing sarcoma [5]. The addition of BCL2/BCL2L1 inhibition to olaparib significantly reduced tumor growth in mouse xenograft models.

Recognizing the high rate of drug resistance in relapsed Ewing sarcoma, recent studies are reporting efforts to prospectively attempt to predict mechanisms of drug resistance to targeted agents in an attempt to prevent or circumvent escape. Cyclin-dependent kinase (CDK) 4/6 inhibitors demonstrate promising activity in Ewing sarcoma, and Guenther et al recently sought to predict candidate genes mediating resistance to CDK4/6 inhibition. They showed that Ewing cell resistance to CDK inhibition can be combatted by concomitant targeting of the IGF pathway and propose this dual-targeting approach could be clinically beneficial [6].

In 2018, a phase I study opened testing the clinical analogue (Seclidemstat) of the lysine demethylase 1A (KDM1A/LSD1) inhibitor SP-2509 (Salarius Pharmaceuticals) in Ewing sarcoma. KDM1A is another promising target in the treatment of Ewing sarcoma, as its inhibition disrupts the EWS/FLI1 transcriptional program [7]. Despite SP-2509 showing excellent preclinical single agent efficiency, Pishas and Lessnick recognized the concern for the development of resistance to KDM1A inhibition. Thus, in the current work, the authors sought to prospectively examine the mechanisms of Ewing sarcoma tumor cell resistance to SP-2509. Pishas et al determined that *de novo KDM1A* mutations are not present and do not explain A673 cell resistance to SP-2509. Interestingly, during the initial development of SP-2509 drug resistant (DR) cell lines, Pishas et al discovered that only the Ewing sarcoma cell line A673 could withstand long term drug exposure out of all the Ewing lines tested. This observation highlights the very significant heterogeneity in the response of Ewing tumors and commonly used Ewing cell lines to manipulation of EWS/FLI1 expression and function. SP-2509 treatment results in enhanced expression of proteins such as zyxin, a regulator of the actin cytoskeleton that is usually repressed by EWS/FLI1, and cell morphologic changes observed in A673 SP-2509 DR cells are able to be reversed within a week of drug removal. In stark contrast to the rapidly reversible morphologic change induced by SP-2509, the altered transcriptomic profiles induced by SP-2509 largely persist even following long-term drug washout.

So, how might SP-2509 drug resistance be overcome? The current work explored drug resistance from two clinically relevant points in treatment: first, by examining resistant Ewing cell populations during the primary window of SP-2509 treatment (an *in vitro* correlate to non-responders or disease progression on therapy) and second, following SP-2509 washout (an *in vitro* correlate to clinical end of drug therapy). This approach is important to highlight, as these two scenarios resulted in tumor cells displaying significantly different sensitivities to traditional cytotoxic chemotherapy and other agents. Pishas et al determined that in the initial phases of SP-2509 treatment, HDAC inhibitors are potential agents that could be used to target resistant A673 cell populations. In contrast, A673 SP-2509 DR washout cells were not sensitive to HDAC inhibitors but did regain sensitivity to cytotoxic chemotherapy such as vincristine. Pre-clinical testing of drug resistance mechanisms at different points in therapy may help to maximize the clinical benefit of newly tested agents by providing evidence for combination or ‘next-step’ therapy regimens.

After decades of making little progress in clinical outcomes for metastatic and relapsed Ewing sarcoma, prospectively determining drug escape pathways and designing trials to combine targeted therapy with targeted inhibition of resistance mechanisms would be a fresh attempt at improving survival in relapsed Ewing sarcoma patients. Given the enormous heterogeneity noted in this tumor type, clinical trials also need to include repeat tumor sampling at sites of progression in order to determine the mechanism of drug resistance in individual tumors. Overcoming Ewing sarcoma drug resistance will continue to be a formidable obstacle for current and future clinical trials.
